# The Design and Synthesis of a New Class of RTK/HDAC Dual-Targeted Inhibitors

**DOI:** 10.3390/molecules18066491

**Published:** 2013-06-03

**Authors:** Xuan Zhang, Mingbo Su, Yi Chen, Jia Li, Wei Lu

**Affiliations:** 1 Institute of Drug Discovery and Development, Shanghai Engineering Research Center of Molecular Therapeutics and New Drug Development, East China Normal University, 3663 North Zhongshan Road, Shanghai 200062, China; E-Mail: 52113300004@student.ecnu.edu.cn; 2 School of Life Sciences, East China Normal University, 3663 North Zhongshan Road, Shanghai 200062, China; E-Mail: mbsu@mail.shcnc.ac.cn; 3 State Key Laboratory of Drug Research, Shanghai Institute of Materia Medica, SIBS, Chinese Academy of Sciences, Shanghai 201203, China; E-Mails: ychen@mail.shcnc.ac.cn (Y.C.); jli@mail.shcnc.ac.cn (J.L.)

**Keywords:** receptor tyrosine kinases, histone deacetylase, antitumor, synergistic effect, dual inhibitor

## Abstract

Over the years, the development of targeted medicines has made significant achievements. As a typical example, receptor tyrosine kinases (RTK) inhibitors have become important chemotherapy drugs for a variety of cancers. However, the effectiveness of these agents is always hindered by poor response rates and acquired drug resistance. In order to overcome these limitations, several dual-targeted inhibitors with quinazoline core were designed and synthesized. Though these compounds can simultaneously inhibit histone deacetylases (HDAC) as well as RTK, the structure-activity relationship (SAR) is still not clear enough. To further explore this type of dual-targeted inhibitors, a new class of quinazoline derivatives were designed and synthesized. Their activity evaluations include *in vitro* inhibitory activity of HDAC, epidermal growth factor receptor (EGFR) and human epidermal growth factor receptor 2 (HER2). The SAR study indicated that the introduction of polar group such as hydroxamate on the 4-position of the quinazoline core is more likely to provide a potent HDACi/HER2i hybrid rather than HDACi/EGFRi molecule.

## 1. Introduction

For decades, cytotoxic drugs such as paclitaxel and camptothecin have been the mainstay of cancer chemotherapy. However, almost all traditional cytotoxic agents suffer from severe toxicities and other undesirable side effects. In recent years, the development of targeted medicines has made significant achievements. Unfortunately, though these agents can block key regulators of signaling pathways in cancer, multiple compensatory pathways always attenuate pharmacological effect of single target drugs. In addition, poor response rates and acquired drug resistance also represent a significant barrier to widespread use of targeted medicines. More recently, a number of combinatorial therapies have expanded treatment options [1,2,3,4,5], which can directly block several key signaling pathways and create synergistic effect [6,7,8,9]. But in the meantime, these agents can produce adverse effects related to pharmacokinetics, toxicity and patient compliance. Therefore, in order to overcome these barriers, a new strategy for designing a single molecule with antitumor activities to inhibit multiple targets was developed [10,11,12,13,14].

**Figure 1:**
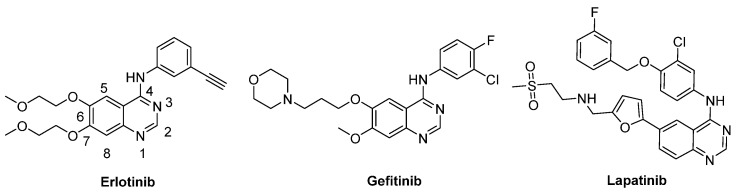
Representative compounds of RTK inhibitors.

Receptor tyrosine kinases (RTK) and histone deacetylases (HDAC) play crucial roles in numerous biological processes. Many selective inhibitors of human epidermal growth factor receptor (HER) family RTK, including erlotinib, gefitinib, and lapatinib, share the same quinazoline core and are important therapeutics against multiple solid tumor cancers (Figure 1) [15,16]. By inducing histone hyperacetylation, HDAC inhibitors (HDACi) can cause growth arrest, differentiation and apoptosis in tumor [17,18,19,20]. Typically, a HDAC inhibitor consists of a capping group, a zinc-binding group (ZBG) and an appropriate linker. There are various ZBGs for HDACi, including hydroxamic acids, carboxylates, aminobenzamides and so on. Up to now, two HDAC inhibitors, vorinostat (SAHA) and *romidepsin* (FK228), have been approved by FDA for the treatment of cutaneous T-cell lymphoma (CTCL) [21,22,23]. However, HDACi monotherapies often have clinical limitations [24]. Recently, several groups investigated a novel type of multi-targeted agents, RTK/HDAC dual inhibitors. Subsequent pharmaceutical study revealed their potential ability to overcome tumour recurrence and drug resistance [8,11,13,25]. In these pioneering studies, the zinc-binding groups such as hydroxamate were all introduced into the hydrophilic segment (6, 7 positions of the quinazoline core). To further explore the structure-activity relationships of these dual action inhibitors, and to find potent antitumor agents, our group initiated a program of RTK/HDAC dual inhibitors.

**Figure 2:**
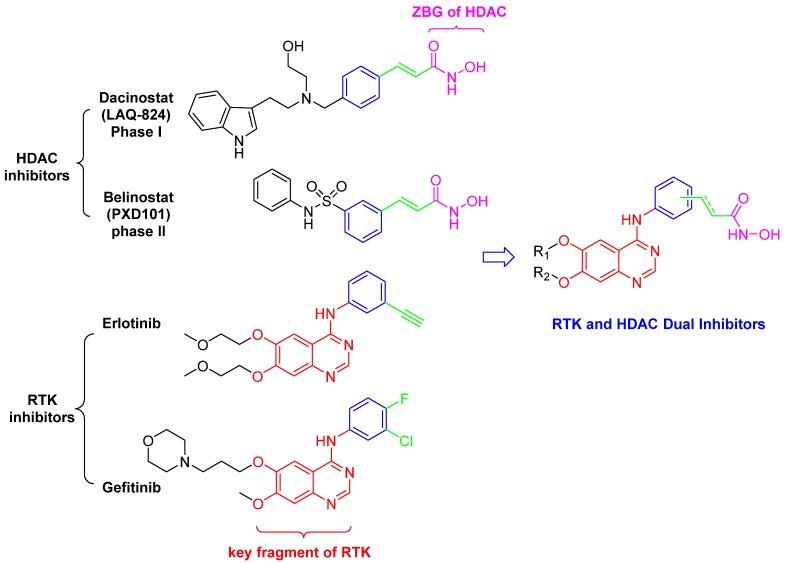
Design of dual inhibitors of RTK and HDAC.

In contrast to the reported RTK/HDAC hybrids, this series of novel dual action inhibitors contain the zinc-binding group on the phenyl ring (Figure 2). To probe the effect of location of ZBG, *para*- and *meta*-substituted compounds were synthesized. Subsequently, we have evaluated their *in vitro* inhibitory activity against HDAC, EGFR and HER2.

## 2. Results and Discussion

### 2.1. Chemisty

**Scheme 1:**
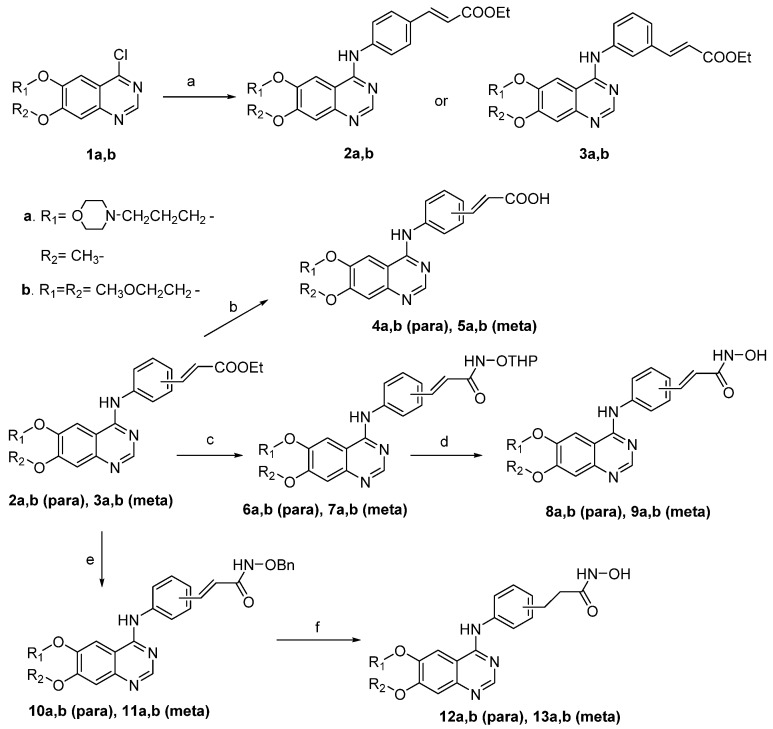
Synthesis of dual-acting HDAC-RTK inhibitors.

The general route for the synthesis of HDAC/RTK dual-acting inhibitors is depicted in Scheme 1. Starting materials **1a**,**b** were synthesized according to the published method [26]. Subsequently, **1a**,**b** were subjected to aromatic nucleophilic substitution with arylamines to give esters **2a**–**b** and **3a-b**, respectively (48%–93% yield). Hydrolysis of these esters proceeded smoothly to afford the corresponding acids **4a**–**b** and **5a**–**b**. Treatment of compounds **2a**–**b** and **3a**–**b** with H_2_N-OTHP in the presence of LHMDS gave the compounds **6a**–**b** and **7a**–**b**, which were hydrolyzed under acidic conditions to afford **8a**–**b** and **9a**–**b**. Similarly, treatment of **2a**–**b** and **3a**–**b** with H_2_N-OBn followed by hydrogenation afforded **12a**–**b** and **13a**–**b**.

*Reagents and conditions*: (**a**) (*E*)-ethyl 3-(4-aminophenyl)acrylate/(*E*)-ethyl 3-(3-aminophenyl)acrylate, *i*-PrOH, reflux; (**b**) LiOH, MeOH, H_2_O, reflux; (**c**) H_2_NOTHP, LHMDS, THF, −78 °C 1h, then 0 °C 5 h; (**d**) 1N HCl (aq), MeOH; (**e**) H_2_NOBn, LHMDS, THF, −78 °C 1 h, then 0 °C 12 h; (**f**) 10% Pd/C, H_2_, MeOH.

### 2.2. Results and Discussion

#### 2.2.1. *In Vitro* HDAC Inhibition

The inhibition of recombinant human HDAC1, HDAC3 and HDAC6 enzymes was tested first, using SAHA as the positive control (Table 1). Generally, most compounds exhibited moderate to good inhibitory activity against HDAC1, HDAC3 and HDAC6 (compounds **8a**–**b**, **9a**–**b**, **12a**–**b** and **13a**–**b**), except for compounds **4a**–**b** and **5a**–**b**, which conformed to the reported information that hydroxamic acid generally showed more potent HDAC inhibitory activity than carboxylic acids [27,28]. In addition, the location of ZBGs also exerted an influence on the HDAC inhibition. Interestingly, the saturated hydroxamates, both *meta*-substituted (**13a**,**b**) and *para*-substituted (**12a**,**b**), showed similar anti-HDAC activity, despite their different ZBG location. The difference between *meta*- and *para*- substitution might be reduced by the flexibility of the saturated chains. By contrast, the HDAC inhibitory activities were quite different for unsaturated hydroxamates **(**compounds **8a**,**b** and **9a**,**b)**. The *para*-substituted **8a**,**b** exhibited moderate inhibition, while the meta-substituted counterparts (**9a**,**b**) showed much more potent activity, suggesting that appropriate rigid linker benefited the coordination between the ZBGs and the zinc ion of the enzyme. Furthermore, the two different hydrophilic moieties on the quinazoline core exhibited no obvious difference for the HDAC inhibitory activity (**8a** *vs* **8b**, **9a** *vs* **9b**, **12a** *vs* **12b** and **13a** *vs* **13b**). Among all these conjugates, compound **9a** showed most potent anti-HDAC activity (HDAC-1 IC_50_ = 0.16 μm; HDAC-3 IC_50_ = 0.18 μm; HDAC-6 IC_50_ = 0.56 μm).

**Table 1:** *In vitro* HDAC Inhibition.

Compound	HDAC-1	HDAC-3	HDAC-6
	IC_50_ ± SD	IC_50_ ± SD	IC_50_ ± SD
	[μM]	[μM]	[μM]
**4a**	- ^a^	-	-
**4b**	-	-	-
**5a**	-	-	-
**5b**	-	-	-
**8a**	5.57 ± 1.87	2.38 ± 0.38	1.66 ± 0.19
**8b**	-	-	2.77 ± 1.02
**9a**	0.16 ± 0.02	0.18 ± 0.05	0.56 ± 0.06
**9b**	0.29 ± 0.05	0.15 ± 0.02	0.56 ± 0.05
**12a**	2.83 ± 0.65	3.29 ± 0.34	5.71 ± 0.99
**12b**	5.82 ± 1.15	3.90 ± 0.78	7.21 ± 0.76
**13a**	1.03 ± 0.27	2.11 ± 0.34	7.63 ± 0.99
**13b**	2.43 ± 0.50	1.57 ± 0.17	4.82 ± 0.37
SAHA	0.25 ± 0.04	0.17 ± 0.02	0.23 ± 0.06
Lapatinib	-	-	-

*^a^* HDAC inhibition < 50% at 20 μg/mL.

#### 2.2.2. *In Vitro* RTK Inhibition

Subsequently, the *in vitro* inhibitory activities of EGFR and HER2 were assessed by enzyme-linked immunosorbent assay (ELISA) [29], employing lapatinib as the positive control. As shown in Table 2, all of these derivatives showed reduced anti-RTK activity, compared with lapatinib, suggesting that the polar groups such as hydroxamate on the phenyl group exerted negative effects on RTK inhibition [13]. On the contrary, the hydroxamate group on the 6, 7 positions of the quinazoline core could retain their RTK inhibition activity as reported [11,13,25]. In the light of the above results, lipophilic benzamide seemed to be more suitable than hydroxamate to serve as the ZBG on the phenyl ring. Cinnamoyl hydroxamates exhibited more potent inhibition against HER2 (compounds **8a**,**b** and **9a**,**b**). Among all these derivatives, compound **8b** showed most potent anti-EGFR and anti-HER2 activities.

**Table 2:** *In vitro* RTK Inhibition.

Compound	EGFR %Inhibition ^a^	HER2 %Inhibition ^b^
**4a**	6.1	18.2
**4b**	20.1	0
**5a**	20.9	0
**5b**	19.0	2.6
**8a**	63.6	74.0
**8b**	70.1	84.6
**9a**	9.7	47.2
**9b**	8.5	64.2
**12a**	0	0
**12b**	44.6	0.1
**13a**	42.6	0
**13b**	20.4	0
SAHA	0	0
Lapatinib	92.7	92.0

*^a^* Inhibition ratio of EGFR, inhibitor was at 10 μg/mL. *^b^* Inhibition ratio of HER2, inhibitor was at 10 μg/mL.

## 3. Experimental 

### 3.1. General

Melting points were taken on a Fisher-Johns melting point apparatus, are uncorrected and reported in degrees centigrade. ^1^H-NMR spectra and ^13^C-NMR were recorded in CDCl_3_, CD_3_OD, D_2_O and DMSO-*d_6_* on a Bruker DRX-500 (500 MHz) or a Bruker Ascend 400 (400 MHz) using TMS as internal standard. Chemical shifts were reported as δ (ppm) and spin-spin coupling constants as *J* (Hz) values. The mass spectra (MS) were recorded on a Finnigan MAT-95 mass spectrometer. The purity of all tested compounds was established by HPLC to be > 95%. HPLC analyses were performed on an Agilent 1200 series instrument using an Agilent Eclipse XDB-C18 (250 mm × 4.6 mm) column.

### 3.2. Chemistry

#### 3.2.1. General Procedure for the Synthesis of Compounds **2a**, **2b**, **3a**, **3b**

Substituted 4-chloroquinazoline (1.0 eq) and corresponding arylamine (2.0 eq) were refluxed in isopropanol for 5 h. After cooling to room temperature, the mixture was treated with Et_2_O. The resulting solid was filtered and washed with EtOAc.

*Ethyl (E)-3-(4-((7-methoxy-6-(3-morpholinopropoxy)quinazolin-4-yl)amino)phenyl)acrylate* (**2a**). Starting from **1a** and (*E*)-ethyl 3-(4-aminophenyl)acrylate, 48.3% of **2a** was obtained as a white solid according to the aforementioned procedure, mp: 198–200 °C. ^1^H-NMR (500 MHz, CDCl_3_) *δ* 1.34 (t, *J* = 7.1 Hz, 3H), 2.10–2.15 (m, 2H), 2.40–2.50 (m, 4H), 2.58 (t, *J* = 7.1 Hz, 2H), 3.70–3.75 (m, 4H), 4.00 (s, 3H), 4.21 (t, *J* = 6.6 Hz, 2H), 4.27 (q, *J* = 7.1 Hz, 2H), 6.39 (d, *J* = 16.0 Hz, 1H), 7.14 (s, 1H), 7.47(s, 1H), 7.57 (d, *J* = 8.5 Hz, 2H), 7.68 (d, *J* = 16.0, 1H), 7.79 (d, *J* = 8.5 Hz, 2H), 8.69(s, 1H) ; HRMS (ESI): *m/z* calcd for C_27_H_33_N_4_O_5_ (M+H^+^): 493.2446, found: 493.2452.

*Ethyl (E)-3-(4-((6,7-bis(2-methoxyethoxy)quinazolin-4-yl)amino)phenyl)acrylate* (**2b**). Starting from **1b** and (*E*)-ethyl 3-(4-aminophenyl)acrylate, 81.2% of **2b** was obtained as a white solid according to the aforementioned procedure, mp: 233–236 °C. ^1^H-NMR (500 MHz, D_2_O-DMSO-*d_6_*) *δ* 1.20 (t, *J* = 7.2 Hz, 3H), 3.31 (s, 3H), 3.33 (s, 3H), 3.70–3.78 (m, 4H), 4.12 (q, *J* = 7.2 Hz, 2H), 4.18–4.25 (m, 4H), 6.35 (d, *J* = 16.0 Hz, 1H), 7.06 (s, 1H), 7.43–7.60 (m, 5H), 7.75 (s, 1H), 8.53 (s, 1H); HRMS (ESI): *m/z* calcd for C_25_H_30_N_3_O_6_ (M+H^+^): 468.2129, found: 468.2134.

*Ethyl (E)-3-(3-((7-methoxy-6-(3-morpholinopropoxy)quinazolin-4-yl)amino)phenyl)acrylate* (**3a**). Starting from **1a** and (*E*)-ethyl 3-(3-aminophenyl)acrylate, 85.3% of **3a** was obtained as a white solid according to the aforementioned procedure, mp: 104–105 °C. ^1^H-NMR (400 MHz, DMSO) δ1.27 (t, *J* = 7.1 Hz, 3H), 1.92–2.07 (m, 2H), 2.35–2.43 (m, 4H), 2.46–2.49 (m, 2H), 3.55–3.62 (m, 4H), 3.94 (s, 3H), 4.14–4.27 (m, 4H), 6.60 (d, *J* = 16.0 Hz, 1H), 7.20 (s, 1H), 7.41–7.54 (m, 2H), 7.67 (d, *J* = 16.0 Hz, 1H), 7.81–7.90 (m, 2H), 8.06 (s, 1H), 8.48 (s, 1H), 9.55 (s, 1H). HRMS (ESI): *m/z* calcd for C_27_H_33_N_4_O_5_ (M+H^+^): 493.2450, found: 493.2452.

*Ethyl (E)-3-(3-((6,7-bis(2-methoxyethoxy)quinazolin-4-yl)amino)phenyl)acrylate* (**3b**). Starting from **1b** and (*E*)-ethyl 3-(3-aminophenyl)acrylate, 93.6% of **3b** was obtained as a white solid according to the aforementioned procedure, mp: 178–181 °C. ^1^H-NMR (500 MHz, D_2_O-DMSO-*d_6_*) *δ* 1.51 (t, *J* = 7.0 Hz, 3H), 3.62 (s, 3H), 3.63 (s, 3H), 4.00–4.08 (m, 4H), 4.42–4.50 (m, 6H), 6.70 (d, *J* = 15.8 Hz, 1H), 7.31 (s, 1H), 7.67–7.70 (m, 2H), 7.77 (d, *J* = 15.8 Hz, 1H), 7.85 (d, *J* = 6.5 Hz, 1H), 7.99 (s, 2H), 8.76(s, 1H); HRMS (ESI): *m/z* calcd for C_25_H_30_N_3_O_6_ (M + H^+^): 468.2129, found: 468.2141.

#### 3.2.2. General Procedure for the Synthesis of Compounds **4a**, **4b**, **5a**, **5b**

Ester (1.0 eq) and LiOH (4.0 eq) were refluxed in a mixture MeOH and water (5:2) for 2h. After cooling to 0 °C, the resulting solid was filtered off and washed with water followed by EtOH.

*(E)-3-(4-((7-methoxy-6-(3-morpholinopropoxy)quinazolin-4-yl)amino)phenyl)acrylic acid* (**4a**). Starting from **2a**, 89.0% of **4a** was obtained as a yellow solid according to the aforementioned procedure, mp: 242–245 °C. ^1^H-NMR (500 MHz, DMSO-*d_6_*) *δ* 2.30–2.35 (m, 2H), 3.01–3.50 (m, 6H), 3.75–3.85 (m, 2H), 3.90–3.95 (m, 2H), 3.99 (s, 3H), 4.35–4.40 (m, 2H), 6.55 (d, *J* = 16.0 Hz, 1H), 7.39 (s, 1H), 7.61 (d, *J* = 16.0 Hz, 1H), 7. 78 (d, *J* = 8.5 Hz, 2H), 7.87 (d, *J* = 8.5 Hz, 2H), 8.59 (s, 1H), 8.85(s, 1H); HRMS (ESI): *m/z* calcd for C_25_H_29_N_4_O_5_ (M+H^+^): 465.2132, found: 465.2156.

*(E)-3-(4-((6,7-bis(2-methoxyethoxy)quinazolin-4-yl)amino)phenyl)acrylic acid* (**4b**). Starting from **2b**, 65.0% of **4b** was obtained as a yellow solid according to the aforementioned procedure, mp: 298–300 °C (decomp.). ^1^H-NMR (500 MHz, DMSO-*d_6_*) *δ* 3.34 (s, 3H), 3.35 (s, 3H), 3.70–3.78 (m, 4H), 4.35–4.40 (m, 4H), 6.42 (d, *J* = 16.0 Hz, 1H), 7.20 (s, 1H), 7.34 (d, *J* = 16.0 Hz, 1H), 7.56 (d, *J* = 8.5 Hz, 2H), 7.87 (d, *J* = 8.5 Hz, 2H), 7.94 (s, 1H), 8.46 (s, 1H), 9.69 (s, 1H); HRMS (ESI): *m/z* calcd for C_23_H_26_N_3_O_6_ (M + H^+^): 440.1816, found: 440.1867.

*(E)-3-(3-((7-methoxy-6-(3-morpholinopropoxy)quinazolin-4-yl)amino)phenyl)acrylic acid* (**5a**). Starting from **3a**, 80.1% of **5a** was obtained as a white solid according to the aforementioned procedure, mp: 195–197 °C. ^1^H-NMR (500 MHz, DMSO-*d_6_*) *δ* 2.30–2.37 (m, 2H), 3.07–3.17 (m, 2H), 3.30–3.35 (m, 2H), 3.45–3.53 (m, 2H), 3.78–3.85 (m, 2H), 3.92–3.98 (m, 2H), 4.00 (s, 3H), 4.35–4.42 (m, 2H), 6.55 (d, *J* = 16.0 Hz, 1H), 7.41 (s, 1H), 7.50 (t, *J* = 8.0 Hz, 1H), 7.53–7.62 (m, 2H), 7.80 (d, *J* = 8.0 Hz, 1H), 8.07 (s, 1H), 8.61 (s, 1H), 8.84(s, 1H), 11.05–11.15 (br, 1H), 11.76 (s, 1H); HRMS (ESI): *m/z* calcd for C_25_H_29_N_4_O_5_ (M+H^+^): 465.2132, found: 465.2134.

*(E)-3-(3-((6,7-bis(2-methoxyethoxy)quinazolin-4-yl)amino)phenyl)acrylic acid* (**5b**). Starting from **3b**, 70.0% of **5b** was obtained as a white solid according to the aforementioned procedure, mp: 219–222 °C. ^1^H NMR (500 MHz, DMSO-*d_6_*) *δ* 3.35 (s, 3H), 3.37 (s, 3H), 3.70–3.85 (m, 4H), 4.25–4.32 (m, 4H), 6.51 (d, *J* = 15.8 Hz, 1H), 7.23 (s, 1H), 7.40–7.45 (m, 2H), 7.60 (d, *J* = 15.8 Hz, 1H), 7.88 (s, 2H), 8.03 (s, 1H), 8.46 (s, 1H), 9.53 (s, 1H), 11.5–12.5 (br, 1H); HRMS (ESI): *m/z* calcd for C_23_H_26_N_3_O_6_ (M + H^+^): 440.1816, found: 440.1840.

#### 3.2.3. General Procedure for the Synthesis of Compounds **6a**, **6b**, **7a**, **7b**

Ester (1.0 eq) and *O*-(tetrahydro-2*H*-pyran-2-yl)hydroxylamine (3.0 eq) were dissolved in THF. The mixture was cooled to −78 °C and 1 M LHMDS in THF (6.0 eq) was dropped into the flask. After stirring at this temperature for 1 h, it was warmed to 0 °C and reacted for another 5 h. Subsequently it was extracted with EtOAc and concentrated *in vacuo*. The crude product was crystallized in a mixture of EtOAc and petroleum ether and used directly in the next step.

#### 3.2.4. General Procedure for the Synthesis of Compounds **8a**, **8b**, **9a**, **9b**

Compound **6a/6b/7a/7b** in MeOH was treated with 1 M HCl aq (10 eq). The mixture was stirred at room temperature overnight and the solvent was removed under reduced pressure. Crystallization of the crude product with EtOH gave the pure title compounds.

*(E)-N-hydroxy-3-(4-((7-methoxy-6-(3-morpholinopropoxy)quinazolin-4-yl)amino)phenyl)acrylamide* (**8a**). Starting from **6a**, 39.2% of **8a** was obtained as a yellow solid according to the aforementioned procedure, mp: 222–224 °C. ^1^H-NMR (500 MHz, DMSO-*d_6_*) *δ* 2.28–2.35 (m, 2H), 3.09–3.50(m, 6H), 3.70–3.80 (m, 2H), 3.90–3.95 (m, 2H), 3.99 (s, 3H), 4.35–4.40 (m, 2H), 6.49 (d, *J* = 15.8 Hz, 1H), 7.39 (s, 1H), 7.46 (d, *J* = 15.8 Hz, 1H), 7.62–7.87 (m, 4H), 8.56 (s, 1H), 8.83 (s, 1H), 10.80–10.95 (m, 2H), 11.55–11.62 (br, 1H); HRMS (ESI): *m/z* calcd for C_25_H_30_N_5_O_5_ (M + H^+^): 480.2241, found: 480.2254.

*(E)-3-(4-((6,7-bis(2-methoxyethoxy)quinazolin-4-yl)amino)phenyl)-N-hydroxyacrylamide* (**8b**). Starting from **6b**, 65.8% of **8b** was obtained as a yellow solid according to the aforementioned procedure, mp: 237–240 °C. ^1^H-NMR (500 MHz, DMSO-*d_6_*) *δ* 3.35 (s, 6H), 3.45–3.55 (m, 4H), 4.30–4.40 (m, 4H), 6.50 (d, *J* = 15.0 Hz, 1H), 7.38 (s, 1H), 7.48 (d, *J* = 15.0 Hz, 1H), 7.60–7.85 (m, 4H), 8.37 (s, 1H), 8.83 (s, 1H), 10.80–10.85 (br, 1H), 11.41 (s, 1H); HRMS (ESI): *m/z* calcd for C_23_H_27_N_4_O_6_ (M+H^+^): 455.1925, found: 455.1943.

*(E)-N-hydroxy-3-(3-((7-methoxy-6-(3-morpholinopropoxy)quinazolin-4-yl)amino)phenyl)acrylamide* (**9a**). Starting from **7a**, 86.2% of **9a** was obtained as a white solid according to the aforementioned procedure, mp: 230–233 °C. ^1^H-NMR (500 MHz, DMSO-*d_6_*) *δ* 2.30–2.35 (m, 2H), 3.08–3.15 (m, 2H), 3.30–3.50 (m, 4H), 3.78–3.83 (m, 2H), 3.92–3.96 (m, 2H), 3.98 (s, 3H), 4.34–4.40 (m, 2H), 6.54 (d, *J* = 15.5 Hz, 1H), 7.37–7.50 (m, 4H), 7.70–7.75 (m, 1H), 7.92 (s, 1H), 8.57 (s, 1H), 8.83 (s, 1H), 10.80–11.10 (m, 2H), 11.73 (br, 1H); HRMS (ESI): *m/z* calcd for C_25_H_30_N_5_O_5_ (M + H^+^): 480.2241, found: 480.2244.

*(E)-3-(3-((6,7-bis(2-methoxyethoxy)quinazolin-4-yl)amino)phenyl)-N-hydroxyacrylamide* (**9b**). Starting from **7b**, 90.8% of **9b** was obtained as a white solid according to the aforementioned procedure, mp: 214–217 °C. ^1^H-NMR (500 MHz, DMSO-*d_6_*) *δ* 3.35 (s, 6H), 3.68–3.78 (m, 4H), 4.30–4.38 (m, 4H), 6.52 (d, *J* = 16.0 Hz, 1H), 7.35–7.70 (m, 5H), 7.86 (s, 1H), 8.25–8.35 (m, 1H), 8.81 (s, 1H), 10.80–10.90 (br, 1H), 11.25–11.45 (m, 1H); HRMS (ESI): m/z calcd for C_23_H_27_N_4_O_6_ (M+H^+^): 455.1925, found: 455.1933.

#### 3.2.5. General Procedure for the Synthesis of Compounds **10a**, **10b**, **11a**, **11b**

Ester (1.0 eq) and *O*-benzylhydroxylamine hydrochloride (3.0 eq) were dissolved in THF. The mixture was cooled to −78 °C and 1M LHMDS in THF (10.0 eq) was dropped in. After stirring at this temperature for 1 h, it was warmed to room temperature and reacted for another 12 h. Then it was extracted with DCM and concentrated *in vacuo*. The crude product was crystallized in a mixture of EtOAc and petroleum ether and used directly in the next step.

#### 3.2.6. General Procedure for the Synthesis of Compounds **12a**, **12b**, **13a**, **13b**

A mixture of compound **10a/10b/11a/11b** and 10% Pd/C in MeOH was hydrogenated at room temperature for 48 h. The catalyst was then filtered off and the filtrate was concentrated *in vacuo*. Crystallization of the crude product from EtOH gave the pure title compounds.

*N-hydroxy-3-(4-((7-methoxy-6-(3-morpholinopropoxy)quinazolin-4-yl)amino)phenyl)propanamide* (**12a**). Starting from **10a**, 36.6% of **12a** was obtained as a white solid according to the aforementioned procedure, mp: 155–157 °C. ^1^H-NMR (400 MHz, DMSO-*d_6_*) δ 1.94–2.06 (m, 2H), 2.29 (t, *J* = 7.4 Hz, 2H), 2.36–2.48 (m, 6H), 2.82 (t, *J* = 7.4 Hz, 2H), 3.53–3.63 (m, 4H), 3.93 (s, 3H), 4.18 (m, 2H), 7.10–7.26 (m, 3H), 7.65 (d, *J* = 8.1 Hz, 2H), 7.83 (s, 1H), 8.41 (s, 1H), 8.68 (s, 1H), 9.40 (s, 1H), 10.37 (s, 1H). HRMS (ESI): *m/z* calcd for C_23_H_32_N_5_O_5_ (M + H^+^): 482.2398, found: 482.2405.

*3-(4-((6,7-bis(2-methoxyethoxy)quinazolin-4-yl)amino)phenyl)-N-hydroxypropanamide* (**12b**). Starting from **10b**, 15.9% of **12b** was obtained as a white solid according to the aforementioned procedure, mp: 188–191 °C. ^1^H-NMR (400 MHz, DMSO-*d_6_*) δ 2.28 (t, *J* = 7.6 Hz, 2H), 2.82 (t, *J* = 7.6 Hz, 2H), 3.35 (s, 3H), 3.37 (s, 3H), 3.69–3.84 (m, 4H), 4.23–4.35 (m, 4H), 7.14–7.26 (m, 3H), 7.67 (d, *J* = 7.9 Hz, 2H), 7.85–7.99 (m, 1H), 8.41 (s, 1H), 8.68 (s, 1H), 9.41 (br, 1H), 10.37 (s, 1H). HRMS (ESI): *m/z* calcd for C_23_H_29_N_4_O_6_ (M+H^+^): 457.2082, found: 457.2087.

*N-hydroxy-3-(3-((7-methoxy-6-(3-morpholinopropoxy)quinazolin-4-yl)amino)phenyl)propanamide* (**13a**). Starting from **11a**, 52.1% of **13a** was obtained as a pale yellow solid according to the aforementioned procedure, mp: 118–120 °C. ^1^H-NMR (400 MHz, DMSO-*d_6_*) δ 1.92–2.08 (m, 2H), 2.26–2.34 (m, 2H), 2.36–2.48 (m, 6H), 2.84 (t, *J* = 7.6 Hz, 2H), 3.53–3.65 (m, 4H), 3.93 (s, 3H), 4.19 (t, *J* = 6.3 Hz, 2H), 6.96 (d, *J* = 7.2 Hz, 1H), 7.18 (s, 1H), 7.29 (t, *J* = 7.8 Hz, 1H), 7.56 (s, 1H), 7.69 (d, *J* = 8.5 Hz, 1H), 7.84 (s, 1H), 8.39–8.48 (m, 1H), 8.68 (s, 1H), 9.40 (s, 1H), 10.37 (s, 1H). HRMS (ESI): *m/z* calcd for C_23_H_32_N_5_O_5_ (M+H^+^): 482.2398, found: 482.2410.

*3-(3-((6,7-bis(2-methoxyethoxy)quinazolin-4-yl)amino)phenyl)-N-hydroxypropanamide* (**13b**). Starting from **11b**, 39.8% of **13b** was obtained as a pale yellow solid according to the aforementioned procedure, mp: 119–122 °C. ^1^H-NMR (500 MHz, CD_3_OD) *δ* 2.46 (t, *J* = 7.5 Hz, 2H), 3.01 (t, *J* = 7.5 Hz, 2H), 3.49 (s, 3H), 3.50 (s, 3H), 3.87–3.92 (m, 4H), 4.35–4.43 (m, 4H), 7.23 (d, *J* = 7.5 Hz, 1H), 7.27 (s, 1H), 7.42 (t, *J* = 7.5 Hz, 1H), 7.52–7.60 (m, 2H), 8.03 (s, 1H), 8.67 (s, 1H); HRMS (ESI): *m/z* calcd for C_23_H_29_N_4_O_6_ (M+H^+^): 457.2082, found: 457.2080.

### 3.3. Evaluation of Enzyme Activities

#### 3.3.1. HDAC Enzymatic Assay *in Vitro*

Recombinant human HDAC1, HDAC3 and HDAC6 were cloned and expressed in insect High5 cells using the baculovirus expression system, and purified using Ni-NTA (QIAGEN). The inhibitory activity of HDAC1 and HDAC3 was determined with the HDAC substrate (Ac-Lys-Tyr-Lys (ε-acetyl)-AMC) while the anti-HDAC6 activity was assayed with another HDAC substrate (Boc-Lys (ε-acetyl)-AMC). The reaction was carried out in black 384-well plates (OptiPlateTM-384F, PerkinElmer) at room temperature. The typical inhibition assay was carried out in 25 μl system containing 25 mM Hepes, 137 mM NaCl, 2.7 mM KCl and 4.9 mM MgCl_2_, pH 8.0, HDAC protein (20–200 nM), HDAC substrate (5–50 μM) and 20 μg/mL individual compounds. Positive controls contained all the above components except the inhibitor. The negative controls contained neither enzyme nor inhibitor. After incubated for 24 h and 3 h respectively, the reaction of HDAC1, HDAC3 and HDAC6 was quenched with the addition of 25 l Trypsin (diluted to final concentration 0.3125%). The plates were incubated for 30 min at room temperature to allow the fluorescence signal to develop. The fluorescence generated was monitored at a 355nm (excitation) and 460nm (emission) in the EnVision multilabel plate reader (PerkinElmer Life Sciences, Boston, MA, USA).

#### 3.3.2. EGFR and HER2 Inhibition Assay

EGFR and HER2 kinase activity were assessed using HTScan EGFR and HER2 kinase assay kits (Cell Signaling Technology, Danvers, MA, USA). In short, the GST-EGFR fusion protein was incubated with synthetic biotinylated peptide substrate and 10 μg/mL inhibitors in the presence of 400 μM ATP. Phosphorylated substrate was captured with strapavidin-coated 96-well plates. The level of phosphorylation was monitored by anti-phosphotyrosine and europium-labeled secondary antibodies (DELFIA, Perkin-Elmer). The enhancement solution was added at the end of the assay and enzyme activity was measured in the Wallac Victor II 1420 microplate reader at 615 nM.

## 4. Conclusions

In summary, a new series of quinazoline derivatives was designed based on the reported synergistic effect between RTK and HDAC inhibitors. These hybrids featured the key moieties of *gefitinib* or *erlotinib* as well as the ZBG of HDACi. The inhibitory activities against HDAC, EGFR and HER2 under cell-free conditions were evaluated. Almost all hybrids with hydroxamate segment exhibited potent HDAC inhibitory activity. In addition, the appropriate rigid linker facilitated the binding of hydroxamate segment with zinc ion at the bottom of the active site. Compared with SAHA, compound **9a** showed similar anti-HDAC activities against HDAC1 (IC_50_ = 0.16 μm), HDAC3 (IC_50_ = 0.18 μm) and HDAC6 (IC_50_ = 0.56 μm). However, these hydrids exhibited reduced inhibitory activities against EGFR and HER2. The primary SAR study indicated that the introduction of polar group such as hydroxamate on the 4-position of the quinazoline core is more likely to provide a potent HDAC/HER2 dual inhibitor rather than HDACi/EGFRi hybrid molecule. To further improve anti-RTK activity, lipophilic ZBG such as aminobenzamide would be worthwhile investigating. The in-depth SAR analysis provided concrete foundations for future optimization.
